# Successful Management of Right Ventricular Failure After Emergent Transcatheter Mitral Valve Edge-to-Edge Repair With Inhaled Nitric Oxide: A Case Report

**DOI:** 10.7759/cureus.45469

**Published:** 2023-09-18

**Authors:** Yuma Sato, Akio Yanagi, Shinichi Kakumoto, Hiroshi Miyawaki

**Affiliations:** 1 Department of Anesthesiology, Kokura Memorial Hospital, Kitakyusyu, JPN

**Keywords:** pulmonary vascular resistance, pulmonary hypertension, transcatheter mitral valve edge-to-edge repair, right ventricular failure, inhaled nitric oxygen

## Abstract

Mitral regurgitation (MR) induces left ventricular failure and pulmonary hypertension (PH) and can lead to right ventricular (RV) failure. Inhaled nitric oxide (iNO) decreases pulmonary vessel resistance. iNO has been used in patients with PH and RV failure.

We present a case with cardiogenic shock due to severe degenerative MR. The patient underwent emergent transcatheter mitral valve edge-to-edge repair (TEER). Despite TEER had been successfully performed, hemodynamics did not improve due to RV failure. Administration of iNO improved hemodynamics. This case suggests administration of iNO could be an effective option for RV failure after TEER.

## Introduction

Mitral regurgitation (MR) elevates left atrial and pulmonary venous pressures. Those elevated pressures might cause disruption of the alveolar-capillary complex and elevation of pulmonary vascular resistance (PVR), resulting in pulmonary hypertension (PH) and right ventricular (RV) failure [1]. Inhaled nitric oxide (iNO) is a selective pulmonary vasodilator and decreases PVR [2]. Therefore, iNO is often useful for treating patients with PH and RV failure [3,4].

Transcatheter mitral valve edge-to-edge repair (TEER) is a minimally invasive option for the treatment of patients with MR who are judged to be inoperable or at high surgical risk [5]. Patients with perioperative RV failure in TEER have high rates of mortality and hospitalization [6]. The effectiveness of iNO for RV failure in TEER has never been reported.

In this case report, we present a case in which iNO was an effective option for residual RV failure after the emergent TEER in a patient with cardiogenic shock due to severe degenerative MR.

## Case presentation

A 68-year-old male (height, 168 cm; weight, 60 kg) had chronic heart failure with severe MR, moderate tricuspid regurgitation, and paroxysmal atrial fibrillation.

**Figure 1:**
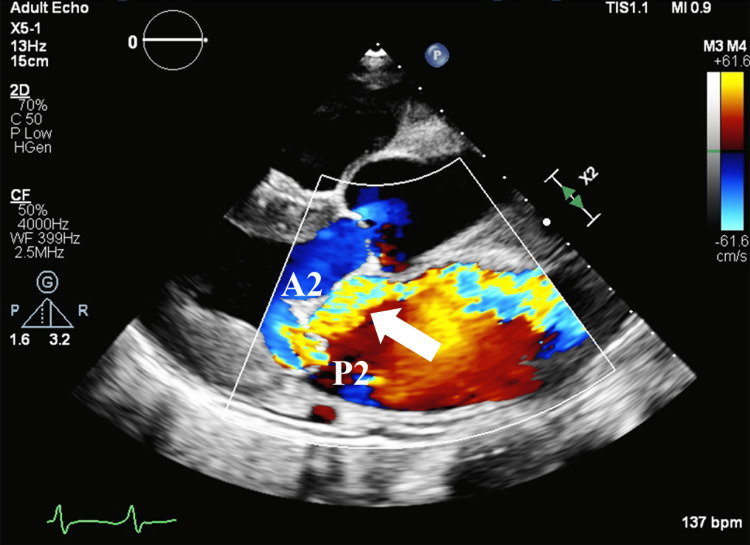
Image of preoperative screening with transthoracic echocardiography There was a mid-posterior mitral leaflet (P2) prolapse with severe MR. The white arrow indicates an MR jet.

The patient had been scheduled for surgical mitral valve repair three years ago for severe MR but refused the intervention and dropped out of the follow-up. The patient was rushed to our hospital due to dyspnea followed by anuria and leg edema caused by exacerbation of heart failure. The patient had no history of kidney disease. When examined in the emergency department, the patient’s blood pressure was 106/75 mmHg, pulse rate was 126/min irregular, and respiratory rate was 22/min with percutaneous oxygen saturation (SpO2) of 99% with 3 L/min of oxygen insufflation. A chest X-ray revealed an enlarged heart and bilateral pleural effusion. A transthoracic echocardiogram showed an LV ejection fraction of 55% (modified Simpson method) and an LV end-diastolic diameter of 56.1 mm without LV wall asynergy. The pathology of MR was prolapse of the mid-posterior mitral leaflet (P2) (Figure 1). Tricuspid annular plane systolic excursion (TAPSE) was reduced to 10.9 mm. The patient had RV failure as well as LV failure. Blood gas analysis revealed a lactate level of 12.0 mmol/L. The patient had cardiogenic shock. When an intra-aortic balloon pumping (IABP) and a pulmonary artery catheter were inserted, pulmonary artery pressure (PAP) was 56/21/37 (systolic/diastolic/mean) mmHg, the mixed venous oxygen saturation (SvO2) was 37%, and pulmonary capillary wedge pressure was 30 mmHg. The patient persisted in experiencing refractory heart failure with cardiogenic shock, despite the prompt implementation of mechanical circulatory support. Therefore, mitral valve intervention was considered necessary. The mortality score calculated by the Society of Thoracic Surgeons score (STS score) was 46.5% and that calculated by the European System for Cardiac Operative Risk Evaluation II (EuroSCORE II) was 38.5%. Surgical mitral valve repair was considered difficult due to the patient's extremely poor general condition. We decided to perform TEER using a MitraClip device under general anesthesia.

**Figure 2:**
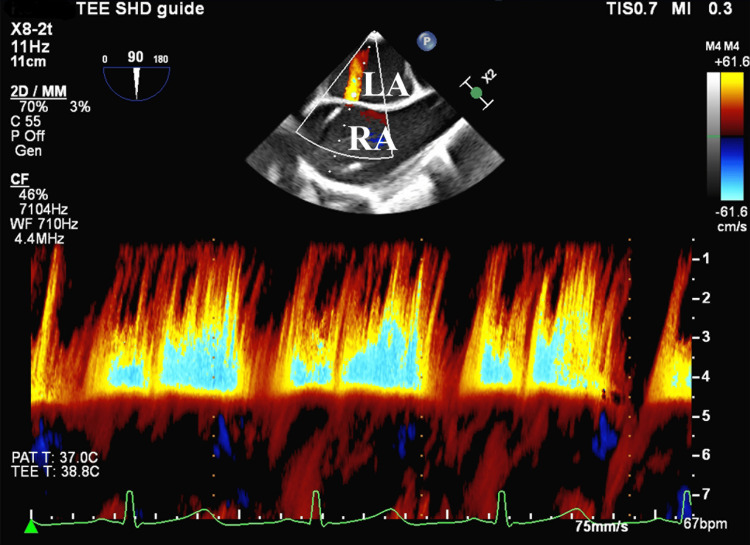
Image of intraoperative screening with transesophageal echocardiography A right-to-left shunt became continuous after removing the steerable guide catheter. LA: left atrium, RA: right atrium

After the introduction of general anesthesia, arterial blood pressure (ABP) dropped from 122/64 to 60/32 mmHg despite the administration of dobutamine (5 mcg/kg/min), milrinone (0.25 mcg/kg/min), and noradrenaline (0.05 mcg/kg/min) and IABP support. PAP was 51/28/40 mmHg and central venous pressure (CVP) was 17 mmHg at that time. The procedure began with the right femoral vein puncture. A slight right-to-left shunt appeared on transesophageal echocardiography after atrial trans-septal puncture. Subsequently, a 24-Fr steerable guiding catheter was inserted into the left atrium. Finally, a clip was implanted in the A2/P2 segment. Transesophageal echocardiography showed that residual MR was mild without mitral stenosis. Although MitraClip was successful, hemodynamics did not improve. Immediately after removing the steerable guiding catheter, a right-to-left shunt developed continuously (Figure 2), and SpO_2_ rapidly decreased from 98% to 90% (F_I_O_2_: 0.4). We assumed that successful TEER reduced left atrial pressure, making the right-to-left shunt continuous. We decided to occlude the iatrogenic atrial septal defect (iASD). Occlusion of the iASD was performed with a 6-mm-diameter Amplatzer cribriform occluder (Abbott Vascular Japan, Tokyo, Japan). When the occlusion of the iASD successful, SpO_2_ increased 94 % to 100% (F_I_O_2_: 0.5). The patient remained in cardiogenic shock (SvO_2_ was 55%) despite inotropic and IABP support.

Transesophageal echocardiography revealed that RV fractional area change was 28%. PAP was 43/25/33 mmHg and CVP was 12 mmHg. PVR was high at 876 dynes・sec・cm^－5^. We calculated the pulmonary artery pulsatility index (PAPi) of 1.5 to complement echocardiographic findings. Despite unloading pulmonary circulation by TEER, the patient still suffered from PH and RV failure. When iNO was administered at 20 ppm through an INOflo DS (Mallinckrodt Pharmaceuticals, Tokyo, Japan) without any other additional anesthetic interventions, hemodynamics improved dramatically in a few minutes. ABP increased from 59/27 to 93/44 mmHg, PAP decreased from 43/25/33 to 36/18/27 mmHg, CVP decreased from 12 to 8mmHg, SvO_2_ increased from 55% to 75%, and cardiac output increased from 2.1 to 3.1 L/min (Table 1).

**Table 1:** Hemodynamic data of post-TEER, after the iASD occlusion, and after iNO administration at 20 ppm Although hemodynamics improved after successful TEER, it worsened again after iASD occlusion. Despite unloading pulmonary circulation by TEER, the patient still suffered from PH and RV failure. When iNO was administered at 20 ppm without any other additional anesthetic interventions, hemodynamics improved dramatically in a few minutes. ABP increased from 59/27 to 93/44 mmHg, PAP decreased from 43/25/33 to 36/18/27 mmHg, CVP decreased from 12 to 8mmHg, SvO_2_ increased from 55% to 75% and CO increased from 2.1 to 3.1 L/min. TEER: transcatheter mitral valve edge-to-edge repair, iASD: iatrogenic atrial septal defect, iNO: inhaled nitric oxide, ABP: arterial blood pressure, PAP: pulmonary artery pressure, CVP: central venous pressure, PAPi: pulmonary artery pulsatility index, SvO_2_: mixed venous oxygen saturation, CO: cardiac output

	After induction of general anesthesia	Post-TEER	After the iASD occlusion	After iNO administration
ABP (mmHg)	60/32	77/36	59/27	93/44
PAP (mmHg)	51/28/40	42/24/32	43/25 /33	36/18 /27
CVP (mmHg)	17	12	12	8
PAPi	1.4	1.5	1.5	2.3
SvO_2 _(%)	42	63	55	75
CO (L/min)	2.1	2.4	2.2	3.1

The patient was transferred from the operating room to the intensive care unit. The patient was withdrawn from IABP support on postoperative day 2. Administration of iNO was stopped, and the patient was extubated on postoperative day 3. The patient was discharged from the intensive care unit on postoperative day 7 without major complications.

## Discussion

PH was redefined in 2022 as a mean PAP higher than 20 mmHg at rest as assessed by right heart catheterization [7]. PH is a recognized risk factor for increased morbidity and mortality after cardiac surgery [8]. Three mechanisms may contribute to PH associated with mitral valve disease. The first mechanism is the backward transmission of elevated left atrial and pulmonary venous pressures into the pulmonary artery. The second is pulmonary arteriolar vasoconstriction induced by pulmonary venous hypertension. The third is morphologic changes in the pulmonary vasculature such as arteriolar muscularization and formation of neointima and hypertrophy of the distal pulmonary arteries. Although the first two mechanisms seem to be rapidly reversed by a reduction of left atrial and pulmonary venous pressure after mitral valve surgery, the third mechanism is not rapidly reversed [1,9]. Thus, there might be residual high PVR after mitral valve surgery, which would result in RV failure.

Patients with perioperative RV failure in TEER have high rates of mortality and hospitalization due to heart failure [6]. It takes several days to improve RV failure; thus, some patients need inotropes or mechanical circulatory support devices after TEER [10]. Patients undergoing urgent/emergent TEER are more often complicated by cardiogenic shock or RV failure than patients undergoing elective TEER [11,12].

Echocardiographic predictors of RV dysfunction include TAPSE <17 mm, pulsed Doppler S wave <9.5 cm/s, RV fractional area change <35%, RV three-dimensional ejection fraction <45%, and pulsed Doppler RV index of myocardial performance >0.43 [13].

A relatively novel hemodynamic parameter of RV function, PAPi, is defined as the ratio of pulmonary artery pulse pressure to the right atrium pressure. The importance of PAPi in predicting severe RV failure requiring additional hemodynamic support has been reported. However, the normal range of PAPi has not been reported. According to previous reports, receiver operating characteristic curves have demonstrated an optimal PAPi cut-off of <1.85 for RV failure after left ventricular assist device implantation [14] and <1.9 for post-cardiopulmonary bypass RV dysfunction [15].

The right ventricle has a unique crescent shape so each echocardiographic parameter has its own limitations [13]. PAPi is not also perfect because PAPi is influenced by the degree of RV afterload and contractile function and the presence of congestion [16]. A multimodal RV evaluation with echocardiography and hemodynamic parameters enables recognition of RV failure, and early interventions for RV failure might improve outcomes in patients undergoing urgent/emergent TEER.

iNO relaxes pulmonary artery smooth muscle to decrease PVR, which leads to increased cardiac output in patients with RV failure [17]. Other benefits of iNO include rapid onset and offset and a very low incidence of adverse effects in the usual range of doses [2]. We need to pay attention to administering iNO in the case of LV failure. iNO increases left atrial and LV filling so that iNO may cause pulmonary edema in patients with LV failure [18]. In our case, iNO was administered after a successful TEER.

Some studies have reported that iNO is associated with improved RV function, decreased PVR, and optimized ventilation-perfusion matching [3,4]. On the other hand, a meta-analysis of randomized controlled trials of iNO failed to demonstrate its benefit [2]. Despite almost 30 years of application history, there is neither solid evidence nor established consensus for using iNO [19]. The effectiveness of iNO for RV failure in TEER has never been reported. We believe that this report would result in an improvement in clinical outcomes in patients with RV failure after urgent/emergent TEER.

## Conclusions

Patients undergoing urgent/emergent TEER are often complicated by cardiogenic shock or RV failure. Some patients do not improve their hemodynamics due to residual RV failure after urgent/emergent TEER. This is the first report that indicates that the administration of iNO could be an effective option in such cases. Further studies are warranted to validate the safety and efficacy of this strategy.

## References

[1] Patel H, Desai M, Tuzcu EM, Griffin B, Kapadia S (2014). Pulmonary hypertension in mitral regurgitation. J Am Heart Assoc.

[2] Sardo S, Osawa EA, Finco G (2018). Nitric oxide in cardiac surgery: a meta-analysis of randomized controlled trials. J Cardiothorac Vasc Anesth.

[3] Winterhalter M, Simon A, Fischer S (2008). Comparison of inhaled iloprost and nitric oxide in patients with pulmonary hypertension during weaning from cardiopulmonary bypass in cardiac surgery: a prospective randomized trial. J Cardiothorac Vasc Anesth.

[4] Fernandes JL, Sampaio RO, Brandão CM (2011). Comparison of inhaled nitric oxide versus oxygen on hemodynamics in patients with mitral stenosis and severe pulmonary hypertension after mitral valve surgery. Am J Cardiol.

[5] Otto CM, Nishimura RA, Bonow RO (2021). 2020 acc/aha guideline for the management of patients with valvular heart disease: executive summary: a report of the American college of cardiology/american heart association joint committee on clinical practice guidelines. Circulation.

[6] Sugiura A, Shamekhi J, Goto T (2022). Early response of right-ventricular function to percutaneous mitral valve repair. Clin Res Cardiol.

[7] Humbert M, Kovacs G, Hoeper MM (2022). 2022 ESC/ERS guidelines for the diagnosis and treatment of pulmonary hypertension. Eur Heart J.

[8] Nashef SA, Roques F, Michel P, Gauducheau E, Lemeshow S, Salamon R (1999). European system for cardiac operative risk evaluation (EuroSCORE). Eur J Cardiothorac Surg.

[9] Foltz BD, Hessel EA, II II, Ivey TD (1984). The early course of pulmonary artery hypertension in patients undergoing mitral valve replacement with cardioplegic arrest. J Thorac Cardiovasc Surg.

[10] Neuser J, Buck HJ, Oldhafer M (2022). Right ventricular function improves early after percutaneous mitral valve repair in patients suffering from severe mitral regurgitation. Front Cardiovasc Med.

[11] Musuku SR, Mustafa M, Pulavarthi M (2022). Procedural, short-term, and intermediate-term outcomes in propensity-matched patients with severe mitral valve regurgitation undergoing urgent versus elective mitraclip percutaneous mitral valve repair. J Cardiothorac Vasc Anesth.

[12] Lee CW, Huang WM, Tsai YL (2021). Feasibility of the transcatheter mitral valve repair for patients with severe mitral regurgitation and endangered heart failure. J Formos Med Assoc.

[13] Lang RM, Badano LP, Mor-Avi V (2015). Recommendations for cardiac chamber quantification by echocardiography in adults: an update from the American Society of Echocardiography and the European Association of Cardiovascular Imaging. J Am Soc Echocardiogr.

[14] Morine KJ, Kiernan MS, Pham DT, Paruchuri V, Denofrio D, Kapur NK (2016). Pulmonary artery pulsatility index is associated with right ventricular failure after left ventricular assist device surgery. J Card Fail.

[15] Rong LQ, Rahouma M, Neuburger PJ (2020). Use of pulmonary artery pulsatility index in cardiac surgery. J Cardiothorac Vasc Anesth.

[16] Zern EK, Wang D, Rambarat P (2022). Association of pulmonary artery pulsatility index with adverse cardiovascular events across a hospital-based sample. Circ Heart Fail.

[17] Beck JR, Mongero LB, Kroslowitz RM (1999). Inhaled nitric oxide improves hemodynamics in patients with acute pulmonary hypertension after high-risk cardiac surgery. Perfusion.

[18] Loh E, Stamler JS, Hare JM, Loscalzo J, Colucci WS (1994). Cardiovascular effects of inhaled nitric oxide in patients with left ventricular dysfunction. Circulation.

[19] Ichinose F, Zapol WM (2017). Inhaled pulmonary vasodilators in cardiac surgery patients: correct answer is "NO". Anesth Analg.

